# Editorial: Tumor Cell Metabolism and Autophagy as Therapeutic Targets

**DOI:** 10.3389/fonc.2020.573343

**Published:** 2020-12-16

**Authors:** Nadia Jacobo-Herrera, Luis Gomez-Quiroz, Carlos Pérez-Plasencia

**Affiliations:** ^1^ Unidad de Bioquímica, Instituto Nacional de Ciencias Médicas y Nutrición Salvador Zubirán (INCMNSZ), Mexico City, Mexico; ^2^ Laboratorio de Fisiología Celular y Biología Molecular, Departamento de Ciencias de la Salud, Universidad Autónoma Metropolitana-Iztapalapa, Mexico City, Mexico; ^3^ Laboratorio de Genómica, Unidad de Investigación Biomédica en Cáncer, Facultad de Estudios Superiores Iztacala, Universidad Nacional Autónoma de México & Instituto Nacional de Cancerología, Mexico City, Mexico

**Keywords:** cancer metabolic phenotype, autophagy, lncRNAs, microRNAs, cancer metabolic remodeling

Cancer is a group of alterations in the normal functioning of a cell. As is known, the tumor cell has adaptations to its environment that allow it to proliferate in conditions unfavorable to a normal cell. Within these changes is the so-called aberrant metabolism, which permits the tumor cell to continue with its replication rate, avoid programmed cell death, or escape from the immune system cells. Although Otto Warburg first described in 1924 that tumor cells employ glycolysis to produce ATP instead of oxidizing glucose in the tricarboxylic acid (TCA) cycle, it was not until recently that tumor metabolism has gained the attention of the scientific community and studied in detail. The seminal work of the Nobel Prize winner Otto Warburg was a pioneer in the realization not only of glycolysis but also in the description of the deregulation of various enzymes involved in the glycolytic cleavage and concluded with the activation of the enzyme lactate dehydrogenase A (LDH-A).

Recent research has shown that the aberrant metabolism of cancer is not only involved in maintaining a high proliferative rate, but it is also necessary to escape from the stress produced by the hypoxic environment to avoid programmed cell death. The high levels of lactate in the tumor microenvironment prevent the cells of the immune system from reacting efficiently against the incipient tumor growth. Novel findings show that there is a delightful regulation of tumor metabolism exerted by oncogenes, as signaling pathways involved in cell growth and proliferation, such as the phosphoinositide 3-kinase/Akt pathway, actively regulates aerobic glycolysis. Transcriptional networks exert control in tumor metabolic activities; thus, c-Myc activates the expression of LDH-A, a metabolic enzyme that converts pyruvate (the final product of glycolysis) to lactate as well as glutamine synthetase. Hypoxia-inducible transcription factor 1α promotes survival under hypoxic conditions by regulating genes relevant in glycolysis, redox homeostasis, among others. Sterol regulatory element-binding protein transcription factor is involved in the synthesis of fatty acids, activates different enzymes needed to convert acetyl-coenzyme A into fatty acids (1).

There is an exquisite regulatory network that sustains tumor metabolism at both the genetic and epigenetic levels. Non-coding RNAs, such as long non-coding RNAs (lncRNAs) and microRNAs (miRNAs), have been described as managers of the tumor microenvironment, involved in the development of cancer and the regulation of the different hallmarks of cancer. These non-coding molecules are modulators of adaptation to stressful situations such as hypoxia, oxidative stress, and nutrient deprivation.

There are pharmacological strategies designed to inhibit aberrant tumor metabolism. However, the inhibition of enzymes involved in tumor metabolism often causes acute systemic toxicity, injuring enzymes of normal tissues too. Therefore, the possibility of inhibiting metabolic pathways or specific enzymes will depend on whether the systemic blockade is tolerated. Examples of drugs already used in the oncology clinic include inhibitors of DNA synthesis such as antifolates (methotrexate, pemetrexed, and others). Notwithstanding these drugs produce toxicity in normal proliferating tissues like the intestinal epithelium and bone marrow, their use is still valid in therapeutic schemes in different tumors. Therefore, it is an extraordinary opportunity in drug discovery to include novel molecular targets for cancer treatment.

Autophagy is a highly conserved intracellular molecular mechanism in eukaryotes that allows the recycling of proteins and cellular organelles. Several stimuli initiate autophagy; under normal conditions, it starts to degrade misfolded proteins and damaged organelles, depletion of nutrients, embryonic development, or cell death by apoptosis. Although different types of autophagy have been characterized, macroautophagy is the best-studied mechanism and probably the most important in cancer.

The maintenance of cell homeostasis in conditions of nutrient depletion is fundamental to cell survival. At different stages during the development of the tumor mass, conditions of metabolic stress due to starvation are produced, which compromises the viability of the malignant tissue. Moreover, reports mention that hypoxic shock activates autophagy, which plays a central role in chemoresistance (2). On the other hand, activation of autophagy enhances the tumor-associated endothelial cells during the development of new blood vessels supplying the tumor mass (3).

While suppression of glycolysis activates autophagy to recycle nutrients and maintain cell survival, the tumor cell eventually will be able to adapt to its environment and activate other pathways to keep TCA active and thus oxidative phosphorylation. Interestingly, one way to escape the blockage of glycolysis is to raise the metabolism of glutamine and glutamate consumption in different tumors (2). It is essential to recognize that the dual role of autophagy in the promotion of tumor phenotype as a therapeutic target puts in the balance the need for further research in this area to generate therapies that allow the successful treatment of cancer (4).

Cancer metabolism and autophagy have attracted the attention of multiple research centers since the possibility of intervening in their different signaling pathways opens the way for more efficient and specific treatments to diminish toxicity towards normal cells and consequently lessens the repercussions for the patient. The topic is extensive, but thanks to the acceptance of the scientific community, in this Research Topic, we have collected 15 articles, five originals research, and 10 reviews. All of them of high academic quality and with future perspectives that allow a clearer vision about the participation of these metabolic processes in cancer. Following, a brief description of the research works.

In their original research, Sarmiento-Salinas et al. showed that there is a molecular signature based on mitochondrial genes that differentiate basal-like tumors from other molecular subtypes. This gene signature is enriched with genes that participate in reactive oxygen species (ROS) metabolism. Finally, the authors demonstrate that the production of mitochondrial ROS supports the proliferative signaling in triple negative breast cancer whereas its inhibition, using an anti-oxidant agent, induces cells to a decreased proliferation.

Regarding breast cancer treatment, Serrano-Carbajal et al. investigated the metabolic deregulation landscapes in breast cancer (BC) molecular subtypes. They designed through a computation tool, developed by their group, a treatment scheme to regulate purine metabolism independently of the BC subtype with Food and Drug Administration approved drugs in public pharmacological databases.

As above mentioned, tumor cell requires low concentrations of glucose and oxygen, as well as a lactic acidosis medium to survive. Romero-Garcia et al. observed that cell growth is affected depending on the lactic acidosis and the presence or absence of oxygen in the medium. For example, in the cell lines A-427 and MCF-7 in hypoxia conditions, cells do not survive in neutral lactosis but lactic acidosis. On the other hand, they noted that in a lactic acidosis medium, either in normoxia or hypoxia, the mitochondrial mass and mitochondria DNA levels were increased in comparison to neutral lactosis in tumor cells but not in fibroblasts. Therefore, they concluded that lung adenocarcinoma cells induce mitochondrial biogenesis to continue survival and proliferation in lactic acidosis and the absence of glucose.

The discovery of new pharmacological therapies in oncology is a tireless area of research. Natural products have been an inexhaustible source of new molecules that provide chemical and molecular bases for the development of new drugs. In the Couder-Garcia et al. article, the results obtained from the antiproliferative effect of peniocerol in an *in vivo* model of colon cancer are displayed. The mechanism of action of this compound is related to the inhibition of poly (ADP-ribose) polymerase-1 and the decrease in the expression of proliferation cell nuclear antigen (cell proliferation marker) both *in vitro* and in xenografts.

Liu et al. performed a liquid chromatography-mass spectrometry-based methodology to discover potential biomarkers for differential diagnosis of patients with bladder cancer and renal cell carcinoma. The authors found that plasma metabolomics and lipidomics could be useful for the discrimination of the global plasma profiles of the two cancer types evaluated from healthy controls.

The reviews published in this special issue cover several interesting topics. Bermúdez et al. reviewed the lncRNAs related to chemoresistance led by autophagy and the clinical implications of the lncRNAs in colorectal cancer. Autophagy was evaluated by Núñez-Olvera et al. as a therapeutic target in endometrial cancer. Besides, the Acevo-Rodríguez et al.‘s group discussed the crosstalk between translation and autophagy and the implications of this process in tumorigenesis. The review of de la Cruz López et al. covered the importance of the mammalian target of rapamycin complex 1 (mTORC1) in the regulation of mitochondrial metabolism in cancer and also discussed the therapeutic efficacy of mTORC1 inhibitors.

Disturbed glucose metabolism is a characteristic of the cancer cell. The review by Vanhove et al. addressed the importance of glucose pathway, glycolysis, gluconeogenesis, and mitochondrial metabolism in lung cancer, and the importance of understanding all these processes to implement new treatments in the future. Moreover, de la Cruz-López et al. also examined the meaning of the lactate in carcinogenesis and tumor immune evasion, as well as a target for cancer therapy.

Regarding dysregulated metabolism, Pedroza-Torres et al. analyzed the miRNAs and their participation in the tumor cell metabolism and as therapeutic target opportunities in cancer.

The hepatocellular carcinoma (HCC) deserved two reviews. In Che et al.‘s article, the authors focused on the role of the fatty acid synthase (FASN) and the molecular mechanisms involved in the activation of this fatty acid in liver carcinogenesis. Also, put on the table that the inhibition of FASN and related lipogenesis as targets for HCC treatment. The second review by Marquardt and Edlich covers the chronic inflammation and cell death resistance in HCC as two of the main hallmarks of this disease.

Finally, the upcoming field of the role of growth differentiation factor 11 (GDF11) in cancer was analyzed by Simoni-Nieves et al. The growth factor GDF11 has attracted attention in cancer due to its age-related role, targeting mainly the stem cells. This particularity becomes imperative in the tumor cell as they acquire the stemness ability which is reflected in tumor aggressiveness and poor clinical prognosis. The effect of GDF11 is controversial, some authors report tumor suppression active, while others blame the opposite. In this study, the authors discuss the role of GDF11 and its functions known so far in cancer biology and metabolism, including examples in liver, breast, pancreatic, and colorectal cancers, as well as in oral squamous cell and melanoma (7).

In summary, GDF11 is a fascinating component of the transforming growth factor-beta superfamily. Its functions are known to depend on cell progeny, tissue type, degree of differentiation, and even age, which is why its activities are diverse and why its involvement in cancer has become a new area of research.

Ample evidence of the importance of autophagy in the development and maintenance of tumor phenotype is documented. However, controversy arises when autophagy is evaluated from a pro- or antitumor perspective. Along with the development of the tumor mass, autophagy protects the tumor cell from the different attacks it suffers on its progress, hypoxic shock, lack of nutrients, chemotherapeutic agents, radiation, production of reactive oxygen species, to only mention some sources of cellular stress (5). On the other hand, different drugs that promote autophagy and lead to the inhibition of tumor growth have been tested *in vitro* and *in vivo* (6). In both scenarios, it is necessary to deepen on the role played by autophagy either as a tumor growth promoter or as a pharmacological target. Concerning aberrant tumor metabolism, the challenge is to identify enzymes that are expressed exclusively in the tumor cell, contrarily their inhibition may compromise the viability of normal cells and tissues. The identification of lncRNAs and miRNAs that regulate both fundamental processes in cancer biology will have an impact not only on the treatment of this disease but also on the knowledge of the cellular machinery and its involvement in the development of new drugs and personalized medicine.

## Author Contributions

NJ-H and CP-P contributed equally to the conception and writing of the manuscript. All authors contributed to the article and approved the submitted version.

## Funding

Current publication was generated by CONACYT 285884 and UNAM PAPIIT-IN231420.

## Conflict of Interest

The authors declare that the editorial was conducted in the absence of any commercial or financial relationships that could be construed as a potential conflict of interest.

## References

[1] Sun Q Yu X Peng C Liu N Chen W Xu H Activation of SREBP-1c alters lipogenesis and promotes tumor growth and metastasis in gastric cancer. BioMed Pharmacother (2020) 128:110274. 10.1016/j.biopha.2020.110274 32464305

[2] Shiratori R, Furuichi K Yamaguchi M Miyazaki N Aoki H Chibana H Glycolytic suppression dramatically changes the intracellular metabolic profile of multiple cancer cell lines in a mitochondrial metabolism-dependent manner. Sci Rep (2019) 10;9(1):18699. 10.1038/s41598-019-55296-3 PMC6904735 31822748

[3] Kardideh B Samimi Z Norooznezhad F Kiani S Mansouri K Autophagy, cancer and angiogenesis: where is the link? Cell Biosci (2019) 9:65. 10.1186/s13578-019-0327-6 31428311 PMC6693242

[4] García-Castillo V López-Urrutia E Villanueva-Sánchez O Ávila-Rodríguez MÁ. Zentella-Dehesa A Cortés-González C Targeting Metabolic Remodeling in Triple Negative Breast Cancer in a Murine Model. J Cancer (2017) 8(2):178–89. 10.7150/jca.16387 PMC5327367 28243322

[5] Poillet-Perez L Despouy G Delage-Mourrous R Boyer-Guittaut M Interplay between ROS and autophagy in cancer cells, from tumor initiation to cancer therapy. Redox Biol (2015) 184:192. 10.1016/j.redox.2014.12.003 PMC4803791 25590798

[6] Cuomo F Altucci L Cobellis G Autophagy function and dysfunction: potential drugs as anti-cancer therapy. Cancers (2019) 11(10):1464. 10.3390/cancers11101465 PMC6826381 31569540

[7] Sinha M Jang YC Oh J Khong D Wu EY Manohar R Restoring systemic GDF11 levels reverses age-related dysfunction in mouse skeletal muscle. Science (2014) 344:649–52. 10.1126/science.1251152 PMC4104429 24797481

